# Molecular Epidemiology and Antifungal Susceptibility of *Trichophyton* Isolates in Greece: Emergence of Terbinafine-Resistant *Trichophyton* *mentagrophytes* Type VIII Locally and Globally

**DOI:** 10.3390/jof7060419

**Published:** 2021-05-27

**Authors:** Maria Siopi, Ioanna Efstathiou, Konstantinos Theodoropoulos, Spyros Pournaras, Joseph Meletiadis

**Affiliations:** 1 Clinical Microbiology Laboratory, Medical School, “Attikon” University General Hospital, National and Kapodistrian University of Athens, 124 62 Athens, Greece; marizasiopi@hotmail.com (M.S.); ioannaeff@outlook.com (I.E.); spournaras@med.uoa.gr (S.P.); 2 Second Department of Dermatology & Venereology, Medical School, “Attikon” University General Hospital, National and Kapodistrian University of Athens, 124 62 Athens, Greece; theod28@gmail.com

**Keywords:** dermatophytes, *Trichophyton* spp., antifungal resistance, terbinafine, Greece

## Abstract

*Trichophyton* isolates with reduced susceptibility to antifungals are now increasingly reported worldwide. We therefore studied the molecular epidemiology and the in vitro antifungal susceptibility patterns of Greek *Trichophyton* isolates over the last 10 years with the newly released EUCAST reference method for dermatophytes. Literature was reviewed to assess the global burden of antifungal resistance in *Trichophyton* spp. The in vitro susceptibility of 112 *Trichophyton* spp. molecularly identified clinical isolates (70 *T. rubrum*, 24 *T. mentagrophytes*, 12 *T. interdigitale* and 6 *T. tonsurans)* was tested against terbinafine, itraconazole, voriconazole and amorolfine (EUCAST E.DEF 11.0). Isolates were genotyped based on the internal transcribed spacer (ITS) sequences and the target gene squalene epoxidase (SQLE) was sequenced for isolates with reduced susceptibility to terbinafine. All *T. rubrum, T. interdigitale* and *T. tonsurans* isolates were classified as wild-type (WT) to all antifungals, whereas 9/24 (37.5%) *T. mentagrophytes* strains displayed elevated terbinafine MICs (0.25–8 mg/L) but not to azoles and amorolfine. All *T. interdigitale* isolates belonged to ITS Type II, while *T. mentagrophytes* isolates belonged to ITS Type III* (*n* = 11), VIII (*n* = 9) and VII (*n* = 4). All non-WT *T. mentagrophytes* isolates belonged to Indian Genotype VIII and harbored Leu393Ser (*n* = 5) and Phe397Leu (*n* = 4) SQLE mutations. Terbinafine resistance rates ranged globally from 0–44% for *T. rubrum* and 0–76% for *T. interdigitale*/*T. mentagrophytes* with strong endemicity. High incidence (37.5%) of terbinafine non-WT *T. mentagrophytes* isolates (all belonging to ITS Type VIII) without cross-resistance to other antifungals was found for the first time in Greece. This finding must alarm for susceptibility testing of dermatophytes at a local scale particularly in non-responding dermatophytoses.

## 1. Introduction

Superficial mycoses are estimated to affect about 20–25% of the world’s population and their prevalence is increasing [1]. Of note, dermatophyte infections impose a considerable economic burden on the healthcare systems since over $800 million/year are spent on their management [2], excluding indirect costs related to unnecessary testing/medical procedures and inappropriate treatment before a diagnosis is established [3]. Meanwhile, resistance in dermatophytes and particularly in *Trichophyton* spp. has recently emerged as a global public health problem [4]. Terbinafine, a synthetic allylamine derivative that inhibits fungal growth by blocking the activity of squalene epoxidase (SQLE) resulting in the accumulation of squalene and depletion of ergosterol from the fungal wall, is considered as the first-line therapy for tinea infections [5]. *Trichophyton rubrum* clinical isolates resistant to terbinafine are sporadically described in the literature [6,7,8,9]. On the other hand, an outbreak of terbinafine-resistant dermatophytosis has been reported in India in 2018 [10]. While the epidemic of treatment-refractory cases in India is escalating [6], transmission of the terbinafine-resistant *T. mentagrophytes* ITS Type VIII to other countries is becoming a reality due to globalization as such strains are now increasingly reported in several Asian and European countries [11]. Most characteristically, it is estimated that such an isolate is recovered in routine diagnostics about every two to three weeks in Germany [12], while, worryingly, a proportion of them have been reported to exhibit cross-resistance to itraconazole [6,10,12]. Therefore, knowledge of in vitro antifungal susceptibility of dermatophytes is becoming more crucial than ever.

Currently, in vitro susceptibility testing of dermatophytes is characterized by technical complexity discouraging its implementation in laboratory routine, thereby hindering the determination of the actual burden of antifungal resistance [13]. At the same time, the lack of clinical breakpoints and/or epidemiological cut-off (ECOFF) values hampers the clinical application of minimum inhibitory concentration (MIC) data obtained from several non-standardized methods, which is however highly needed in the presence of worldwide spread of antifungal-resistant isolates [4]. Nevertheless, the European Committee on Antimicrobial Susceptibility Testing (EUCAST) has recently released a new method for antifungal susceptibility testing against microconidia-forming dermatophytes, including tentative ECOFFs against *T. rubrum* and *T. interdigitale*, which has been validated in a multicenter setting [14]. Thus, data enrichment with MIC distributions encompassing a large number of geographically diverse *Trichophyton* spp. isolates generated by different laboratories will facilitate the determination of formal ECOFFs that will help to detect non-wild type (WT) isolates and monitor the epidemiology of dermatophytosis.

To date, there are still no studies outlining the antifungal susceptibility of *Trichophyton* spp. determined by the optimized EUCAST standard procedure [14]. Furthermore, published data on the antifungal susceptibility patterns of Greek dermatophyte clinical isolates are lacking, whereas the severe socioeconomic events affecting our country during the last 10 years (prolonged financial crisis and rising tide of refugee and migrant populations from Asia and Africa) may have an impact on their epidemiology. Phylogenetic analysis of clinical isolates has shown considerable variation among *Trichophyton* spp. mainly observed within *T. mentagrophytes*/*interdigitale* complex. Based on these grounds, we described the molecular epidemiology and investigated the in vitro susceptibility profile of *Trichophyton* spp. isolated over the last 10 years to topical and systematically applied antifungals commonly used for the treatment of dermatophytosis following the recently reported EUCAST guidelines in an attempt to gain insight into the contemporary state of antifungal resistance in dermatophytes in Greece.

## 2. Materials and Methods

### 2.1. Fungal Isolates

*Trichophyton* isolates recovered from patients with clinically suspected dermatophytosis attending the outpatient Dermatology-Venereology Department of “Attikon” University General Hospital over the past decade (2010–2019) were tested. Samples were collected according to the standard procedure and were processed for direct microscopic examination using Blankophor in 10% potassium hydroxide. The specimens were inoculated on two plates each of Sabouraud’s dextrose agar supplemented with gentamicin and chloramphenicol (SGC2; bioMérieux) and the other containing phenol red and cyclohexamide (DTM; bioMérieux), which were incubated at 30 °C up to four weeks. Recovered isolates were identified to the genus and species level by standard phenotypic methods based on their colonial and microscopic morphology as well as on their biochemical properties (hydrolysis of urea) [15]. The strains were stored in normal sterile saline with 10% glycerol at −70 °C until the study was performed.

### 2.2. Molecular Identification

Genomic DNA was extracted from fresh fungal cultures subcultured on potato dextrose agar using a column-based method (QIAamp^®^ DNA Mini Kit; Qiagen, Athens, Greece) by combining enzymatic (incubation with protease K at 56 °C for 10 min) and mechanical (10 min vortexing with glass beads) pretreatment. The internal transcribed spacer (ITS)1–5.8S-ITS2 region was amplified using the primer pair ITS1 (5′-TCCGTAGGTGAACCTGCGG-3′) and ITS4 (5′-TCCTCCGCTTATTGATATGC-3′) and polymerase chain reaction conditions were set as previously described [16]. Molecular species identification in all strains was preliminarily performed by restriction fragment length polymorphism (RFLP) analysis of ITS region by MvaI restriction enzyme (Thermo Fisher Scientific, Athens, Greece) revealing distinct recognition band patterns for *T. rubrum* and *T. tonsurans* but not for *T. mentagrophytes* species complex isolates [17], which were subjected to confirmatory molecular identification by Sanger sequencing the ITS region [18]. In the context of the newly introduced taxonomy of dermatophytes, which is built on a molecular multilocus phylogenetic approach, the former *T. mentagrophytes* species complex is differentiated into *T. interdigitale* (anthropophilic) and *T. mentagrophytes* (zoophilic) [19]. Consensus DNA sequences were generated using forward and reverse sequences from ITS primers (DNAStar Lasergene 12 software, DNAStar Inc., Madison, WI, USA) and were compared with the reference sequences deposited in the GenBank database performing nucleotide BLAST searches. Sequence-based species identification was defined by ≥99% sequence similarity with ≥99% query coverage. Phylogenetic relationships were generated with 1000 Bootstrap replication and the Tamura-Nei model as a substitution method (Mega X software, [20]) using ITS sequences retrieved from the GenBank [21,22]. All ITS sequences of the representative isolates were deposited at the GenBank (NCBI, Bethesda, MD, USA).

### 2.3. Antifungal Susceptibility Testing

In vitro susceptibility testing of *Trichophyton* isolates to terbinafine (Sigma-Aldrich, Athens, Greece), itraconazole (Sigma-Aldrich, Athens, Greece), voriconazole (Pfizer Ltd., Kent, UK) and amorolfine (Sigma-Aldrich, Athens, Greece) was performed following the recently proposed EUCAST broth microdilution reference methodology (E.DEF 11.0) [14]. Each inoculum suspension was supplemented with chloramphenicol (PanReac Applichem, Athens, Greece) and cycloheximide (Sigma-Aldrich, Athens, Greece) in a double-strength final concentration of 100 and 600 mg/L, respectively. The final concentrations tested ranged from 0.008 to 8 mg/L for all antifungals, while *Aspergillus flavus* ATCC 204304 and *T. rubrum* SSI-7583 were included as quality control strains in each test run. The MIC endpoints were determined spectrophotometrically (540 nm) as the lowest concentration of drug corresponding to a 50% reduction of the optical density of the drug-free growth control after 5 days of incubation at 25 °C. Isolates exhibiting elevated MIC values above the corresponding tentative ECOFFs of terbinafine, itraconazole, voriconazole and amorolfine for *T. rubrum* (0.03, 0.25, 0.125 and 0.125 mg/L) and *T. interdigitale/T. mentagrophytes* (0.125, 0.25, 1 and 0.5 mg/L)*,* respectively, were tested in duplicate to ensure reproducibility. For *T. tonsurans*, the tentative ECOFFs of *T. interdigitale* were used because of greater genetic similarly and similar MIC distributions than *T. rubrun*. The modal MIC, geometric mean (GM) MIC, MIC_50_ and MIC_90_ (the concentrations that inhibited 50% and 90% of the isolates) were determined for each agent and species. Differences between the log_2_MICs of antifungals were analyzed using one-way analysis of variance followed by Tukey’s multiple comparison tests and a *p*-value < 0.05 was considered statistically significant (GraphPad 7.0 software, San Diego, CA, USA).

### 2.4. Molecular Analysis of the Gene Encoding SQLE

*Trichophyton* strains exhibiting reduced susceptibility to terbinafine were screened for missense mutations in the SQLE gene. Fungal genomic DNA was extracted as described above. The entire gene encoding SQLE was amplified and sequenced as previously described [7]. Sequences were aligned and compared with WT reference sequences retrieved from the GenBank (Mega X software, [20]). All SQLE sequences of the representative isolates were deposited at the GenBank (NCBI, Bethesda, MD, USA).

## 3. Results

### 3.1. Identification

**Figure 1:**
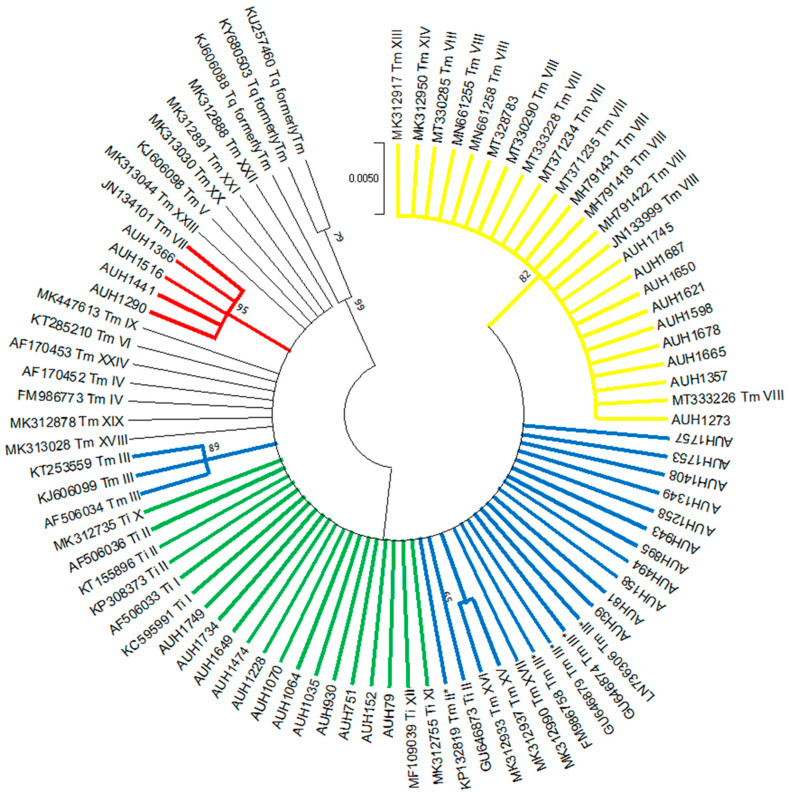
Maximum likelihood phylogenetic tree of *T. interdigitale* (*Ti*) and *T. mentagrophytes* (*Tm*) genotypes based on ITS sequencing. Values at the nodes indicate bootstrap percentages based on 1000 replicates and only branches with bootstrap values above 50% are shown (different clades are highlighted using different colors). ITS sequences of several reference isolates and clinical strains retrieved from the GenBank [21,22] were used for comparative analysis. The isolates of the present study are marked with the prefix AUH.

**Figure 2:**
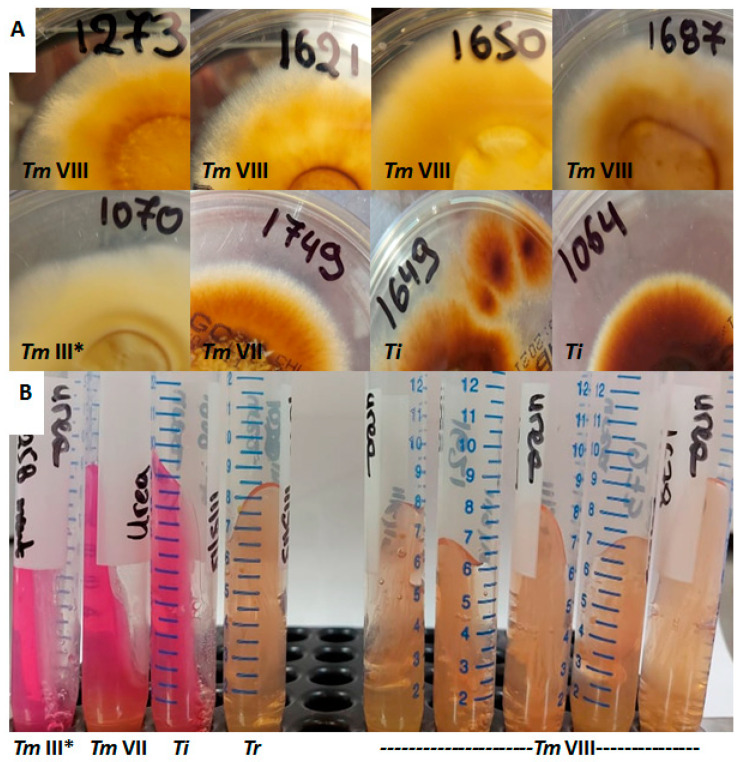
(**A**) Colonies of all but one (Nr.1687) *T. mentagrophytes* ITS Type VIII (*Tm* VIII) isolates had yellowish reverse pigment (upper line), as opposed to *T. interdigitale* (*Ti*) as well as *T. mentagrophytes* Type III* (*Tm* III*) and Type VII (*Tm* VII) isolates (lower line). (**B**) Urease test on Christensen urease agar was positive for *T. interdigitale* and *T. mentagrophytes* ITS Type III* and Type VII, but negative for *T. rubrum* (*Tr*) and *T. mentagrophytes* Type VIII after 7 days of incubation.

Overall, 150 *Trichophyton* isolates were recovered, whereof 38 (25%) failed to grow despite the repeated efforts to revive them from frozen glycerol stocks. RFLP showed the distinct band patterns for *T. rubrum* (*n* = 70)*, T. tonsurans* (*n* = 6) and *T. mentagrophytes*/*T. interdigitale* (*n* = 36) [17]. ITS sequencing and phylogenetic analysis of *T. mentagrophytes*/*T. interdigitale* isolates revealed 12 *T. interdigitale* Type II (GenBank accession no. MW709417-MW709428) and 24 *T. mentagrophytes* (Type III* (*n* = 11), Type VIII (*n* = 9) and Type VII (*n* = 4), GenBank Accession No. MW752105-MW752128) strains (Figure 1). Notably, *T. interdigitale* as well as *T. mentagrophytes* ITS Type III* and Type VII isolates were distributed equally through the years, as opposed to *T. mentagrophytes* Type VIII strains that were isolated during the last two years (2018 *n* = 2, 2019 *n* = 7). Furthermore, *T. mentagrophytes* ITS Type VIII isolates showed distinct macroscopic morphological characteristics. In particular, the reverse of the colonies of the majority of such isolates was yellowish pigmented on SGC2 (8/9 with 1/9 pale brown), in contrast to *T. interdigitale* and *T. mentagrophytes* of other ITS genotypes strains that showed white cream to pale/dark brown pigmentation (Figure 2). In addition, the urease test on Christensen urease agar was positive for *T. interdigitale* and *T. mentagrophytes* ITS Type III* and Type VII after 3–5 days of incubation. On the other hand, *T. rubrum* and *T. mentagrophytes* ITS Type VIII were negative on Christensen urease agar even after 7 days of incubation (Figure 2).

### 3.2. Origin of Isolates

The 112 *Trichophyton* isolates were recovered from skin scrapings (*n* = 52) and nail clippings (*n* = 60) from 109 patients, whereof 65 (60%) were male of median (range, interquartile range) age 55 (0.8–90, 30) years. In particular, 1 (1.0%) episode occurred in an infant patient, 6 (5.5%) in pediatric patients, 60 (55.0%) in adults between 18 and 59 years old, and 42 (38.5%) in elderly patients (≥60 years old). A single isolate was recovered from each patient except two *T. rubrum* strains isolated sequentially (2011 and 2017) from a 68-year-old male patient with tinea unguium (toenails) and three *T. mentagrophytes* strains isolated from serial skin specimens (2018 and 2019) of a 42-year-old female patient with extensive tinea corporis/cruris.

The majority of *T. rubrum* (46/70; 66%) were recovered from nail clippings whereas the majority of *T. mentagrophytes*/*T. interdigitale* (24/36; 67%) isolates was recovered from skin scrapings. All *T. interdigitale* isolates were associated with tinea unguium. On the other hand, *T. mentagrophytes* ITS Type III* and Type VIII isolates caused primarily tinea corporis (10/11; 91%) and tinea cruris (8/9; 89%), respectively, whereas all Type VII strains were identified to be the causative agents of tinea genitalis cases.

### 3.3. Antifungal Susceptibility

Τhe MIC values of quality control strains were reproducible and fell within the established ranges at the target MICs. Table 1 summarizes the susceptibility patterns of isolates tested. For *T. rubrum*, the in vitro MICs of terbinafine were lower compared to those of azoles and amorolfine (*p* < 0.0001). In particularly, terbinafine, voriconazole, itraconazole and amorolfine GM MICs (MIC ranges) were 0.022 (≤0.008–0.03), 0.060 (0.016–0.125), 0.069 (0.016–0.25) and 0.045 (0.06–0.125) mg/L, respectively. Similarly, among all drugs terbinafine MICs were the lowest for both *T. interdigitale* and *T. tonsurans* isolates (*p* < 0.0001 and 0.005, respectively). Namely, terbinafine, voriconazole, itraconazole and amorolfine GM MICs (MIC ranges) were 0.013 (≤0.008–0.03), 0.046 (0.016–0.125), 0.032 (≤0.008–0.06) and 0.064 (0.03–0.125) mg/L, respectively, for *T. interdigitale* and 0.016 (0.016–0.016), 0.097 (0.03–0.25), 0.042 (0.03–0.06) and 0.035 (0.016–0.125) mg/L, respectively, for *T. tonsurans*. On the contrary, there was no statistically significant difference in in vitro antifungal activities among agents tested against *T. mentagrophytes* isolates (*p* = 0.26). In particular, terbinafine, voriconazole, itraconazole and amorolfine GM MICs (MIC ranges) were 0.127 (≤0.008–8), 0.120 (0.016–0.5), 0.065 (0.016–0.25) and 0.176 (0.03–0.5) mg/L, respectively.

### 3.4. Non-WT Phenotypes

All *T. rubrum*, *T. interdigitale* and *T. tonsurans* isolates showed WT phenotype to all antifungals tested. Interestingly, 9/24 (37.5%) *T. mentagrophytes* strains isolated from skin specimens of 7 patients, whereof 5 (71%) were male of median (range, interquartile range) age 42 (0.8–90, 40) years, exhibited reduced susceptibility to terbinafine (MICs 0.25–8 mg/L) but not to azoles and amorolfine. Of note, phylogenetic analysis revealed that all the aforementioned belonged to *T. mentagrophytes* ITS Type VIII. SQLE gene sequencing showed that five isolates had the amino acid change Leu393Ser (TTA→TCA) and four the Phe397Leu (TTC→TTA) (GenBank Accession No. MZ029694-MZ029702), which confer non-WT phenotype to terbinafine. Patient and isolate related data are presented in Table 2.

## 4. Discussion

In the light of continuing emergence of antifungal-resistant dermatophytes worldwide, susceptibility monitoring, specifically in previously non-investigated geographical areas, is warranted. To our knowledge, this is the first laboratory-based study aiming to assess the extent of resistance phenotypes to clinically used oral/topical antifungals against Greek *Trichophyton* spp. clinical isolates. Furthermore, we present for the first time in the published literature in vitro antifungal susceptibility patterns of *Trichophyton* spp. determined using the new EUCAST broth microdilution reference method (E.DEF 11.0). Based on the proposed tentative ECOFFs, *T. rubrum* and *T. interdigitale* isolates exhibited WT phenotypes to all antifungals tested, with terbinafine being the most potent in vitro (*p* < 0.0001). On the other hand, 9/24 (37.5%) *T. mentagrophytes* strains, all belonging to ITS Genotype VIII, harbored single-point mutations leading to amino acid substitutions (Leu393Ser, Phe397Leu) in the SQLE gene and showed reduced susceptibility to terbinafine (MICs 0.25–8 mg/L) without being cross-resistant to azoles and amorolfine.

The current new taxonomy of dermatophytes separates *T. mentagrophytes* from its clonal offshoot *T. interdigitale* [19]. In fact, this delineation is clinically significant since *T. interdigitale* is exclusively anthropophilic mainly recovered from non-inflammatory tinea unguium and tinea pedis cases, as opposed to *T. mentagrophytes* which is predominantly zoophilic and causes inflammatory tinea of other variants. Indeed, all *T. interdigitale* isolates tested in the present study were associated with onychomycosis, while *T. mentagrophytes* strains caused primarily tinea corporis and tinea cruris, as previously described [21,23]. Nevertheless, the differentiation between the two species by conventional diagnostic methods is challenging given the phenotypic variations among isolates, whereas their distinction based on the source of infection remains quite complex when considering the recent outbreak of chronic, relapsing dermatophytosis in India due to the anthroponotic transmission of *T. mentagrophytes* stains. Thus, accurate identification of the implicated fungal pathogen at a species level using molecular diagnostic tools is vital in order to achieve a targeted therapy and better prognosis as well as for surveillance purposes.

Phylogenetic analysis can settle boundaries between the closely related siblings in the former *T. mentagrophytes* species complex as multiple ITS genotypes have been identified within *T. interdigitale* (*n* = 5) and *T. mentagrophytes* (*n* = 21) [18,21,22]. While *T. interdigitale* ITS genotypes do not vary significantly, *T. mentagrophytes* ITS genotypes may be correlated with specific clinical presentation, mode of transmission, geographical distribution and susceptibility profile to standard antifungals [18,21,22]. Therefore, knowledge of the precise ITS genotype, specifically among *T. mentagrophytes* isolates, has considerable clinical and therapeutic consequences in terms of patient management and counselling. To the best of our knowledge, this is the largest study investigating Greek isolates of the two species on genotype level. In total, four ITS genotypes, including one *T. interdigitale* (Type II) and three *T. mentagrophytes* (Type III*, Type VII and Type VIII), were found. Antifungal resistance has not been reported in *T. interdigitale* Type II isolates and their majority (95%) causes tinea pedis as well as tinea unguium [21], both supporting our findings. *T. mentagrophytes* Type III*, which is considered a European genotype [21], was abundant among the population tested and was identified to be the causative agent of tinea corporis (91%) and tinea capitis (9%) cases, while all *T. mentagrophytes* Type VII (Thailand) strains caused tinea genitalis, as previously described [24,25]. Interestingly, the proportions of *T. mentagrophytes* Type III* and Type VIII isolates were almost comparable (46% versus 37.5%). The latter genotype is endemic in India and Iran [6,21], whereas its transmission occurs extensively in families and among people living in particularly clustered, overcrowded communities [11,12,26]. Indeed, two out of seven patients with *T. mentagrophytes* Type VIII infection were immigrants of Iranian and Syrian (resident in a refugee camp) nationality and one patient lived in a Roma camp in Southern Greece. Unfortunately, given the retrospective nature of our study, the biggest part of our patients’ data was not available. Thus, we can only presume that the surge of these infections is the increased frequency of widespread travel, and importantly, the unprecedented rise in the number of asylum seekers and migrants entering our country. Of note, all strains of Indian genotype were isolated during 2018–2019. Two years earlier, Greece experienced an unprecedented influx of refugees fleeing their home countries in the Middle East because of war. Indeed, the pressure of migration waves was so great that the situation was described as resembling a humanitarian crisis, while it is estimated that refugees now constitute nearly 10% of the Greek population [27].

Dermatophytosis treatment with topical antifungal medications may be effective only in mild, localized infections. Common recommendations for systemic therapy include the presence of multiple site/extensive involvement, chronic/recurrent dermatophytosis, tinea capitis, tinea unguium and localized infections unresponsive to topical antifungals. However, there are no well-established guidelines for the dosage and duration of oral agents, which are usually individualized depending on the clinical response [5]. Furthermore, treatment of tinea in specific patient groups requires a cautious approach given their increased propensity for adverse effects [28]. Among the systemic antifungals terbinafine and itraconazole are the preferred agents. Nevertheless, itraconazole has a primarily fungistatic activity and is more prone to adverse effects [5]. Similarly, ample evidence of safety is available for oral terbinafine and itraconazole in both elderly and children, but in cases where systemic treatment is required, terbinafine is preferred [28]. Voriconazole has only been used off-label for refractory cases and given its cost is likely to be favored over posaconazole in resource-poor settings [11,29,30]. Therefore, terbinafine remains the cornerstone in the treatment of tinea infections due to its favorable pharmacological profile and fungicidal properties.

**Figure 3:**
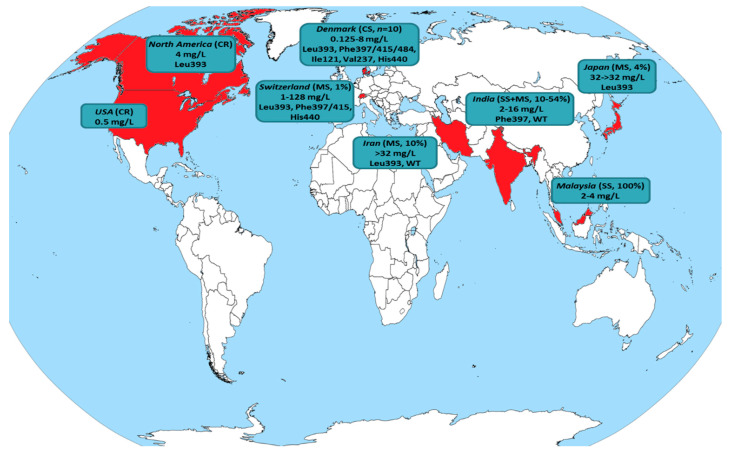
Global distribution of *T. rubrum* isolates exhibiting reduced susceptibility to terbinafine (detailed in Table 3). The country of origin is colored in red, while the type of study (case report: CR, case series: CS, single-center study: SS, multicenter study: MS) along with the recovery rate of isolates displaying non-wild-type (WT) phenotype, terbinafine minimum inhibitory concentrations (mg/L) and amino acid positions with hot-spot mutations in the squalene epoxidase target gene (where available) are presented.

Alarmingly, a recent upsurge of reports revealing terbinafine resistance in *T. rubrum* from Denmark [7], India [6,31], Iran [8] and Japan [32] has raised concerns. Nonetheless, it currently appears that the phenomenon is geographically limited rather than widespread. Figure 3 and Table 3 summarize all published studies where resistance in terbinafine was found for *T. rubrum* clinical isolates. In fact, the majority of data has been obtained through case reports/series (8/18; 44%) and single-center studies (5/18; 28%). Multicenter studies conducted in Switzerland [9] and Japan [32] reported low yields of terbinafine-resistant *T. rubrum* recovery (16/1644; 1% and 5/128; 4%, respectively). On the other hand, three multicenter studies, one from Iran [8] and two carried out in different geographic regions of India [6,31], revealed terbinafine resistance rates ranging from 10% to 44%. However, it should be acknowledged that the number of isolates tested in the aforementioned studies was small (18–20), while studies conducted at similar points in time in the same countries including a larger collection of clinical strains (41–60) showed no *T. rubrum* exhibiting reduced susceptibility to terbinafine [33,34,35]. Hence, the geographical predisposition to differential susceptibility to antifungals, even within the same country, should be taken into account [6]. Furthermore, it is noteworthy that the patients presented in case reports/series (where data are available) responded well to oral azole (itraconazole, fosravuconazole) therapy achieving good clinical outcomes and mycological cure [36,37,38,39]. Similarly, no in vitro cross-resistance to different azoles was demonstrated for strains with a non-WT phenotype to terbinafine [8,32,36,37,38,39,40,41,42,43]. Based on these grounds, azoles may still hold a place in the treatment of terbinafine-resistant *T. rubrum* dermatophytosis.

In Greece, *T. rubrum* is still the predominant species among dermatophytes [51,52], similar to previously published estimates [53]. No terbinafine resistance was found in the present study among Greek *T. rubrum* clinical isolates. Indeed, corresponding data derived from different geographical areas, such as Europe [54,55], Asia [56,57], North [58,59] and South America [60,61], corroborate our finding. Terbinafine overall displayed the highest in vitro activity (terbinafine > amorolfine > voriconazole > itraconazole), which is in line with previous findings [56,60]. Of note, two *T. rubrum* isolates that were obtained sequentially over a span of 7 years from a patient with tinea unguium showed similar in vitro susceptibility profiles (terbinafine, voriconazole, itraconazole and amorolfine MICs 0.03, 0.03, 0.06 and 0.06 mg/L in 2011 and 0.03, 0.06, 0.125 and 0.03 mg/L in 2017, respectively). In this case, the WT phenotypes to all antifungals tested and the recovery of the second isolate several years later advocate that the recurrence of onychomycosis, which occurs at a rate of 20–25% and is highly dependent on host-related as well as environmental factors [19], may not be attributed to acquired antifungal resistance.

On the other hand, the dramatic rise in terbinafine-resistant *T. interdigitale*/*T. mentagrophytes* isolates recovered from all over India within a short period of time is worrisome (Table 4). Single-center studies carried out in several regions around the country located the prevalence of terbinafine-resistant strains between 0% and 74% indicating geographical variation [33,44,45,46,47,62,63]. Indeed, multicenter studies conducted in the southern part of India demonstrated much lower terbinafine resistance rates compared to North India (16% versus 32–76%) [6,10,64]. The fact that India is a large subcontinent with diverse topological and climatic patterns may justify these differences since corresponding studies from different provinces of Iran [21,65] and Switzerland [9,24] showed a narrow range of resistance rates (11% and 0.2–2%, respectively). Importantly, earlier studies have reported *T. interdigitale* as the causative agent of the Indian epidemic of superficial dermatophytosis based on the former taxonomy of fungi [10,46,47,62]. However, on the basis of multigene sequences it is most likely that these strains isolated in different places of India are indeed more closely related to *T. mentagrophytes* ITS Type VIII [18,22,64]. Given their epidemic behavior on humans, it has been recently proposed to classify these terbinafine-resistant Indian strains as a new species named *T. indotineae* based on a polyphasic approach that incorporates molecular as well as distinct morphological and physiological characteristics, among which yellowish reverse pigmentation and no/weak urea hydrolysis, as found in the present study [66,67].

**Figure 4:**
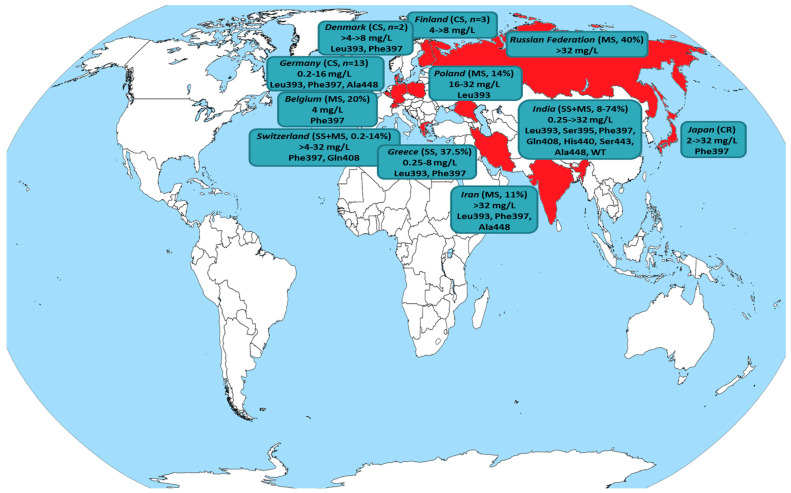
Global distribution of *T. interdigitale*/*T. mentagrophytes* isolates exhibiting reduced susceptibility to terbinafine (detailed in Table 4). The country of origin is colored in red, while the type of study (case report: CR, case series: CS, single-center study: SS, multicenter study: MS) along with the recovery rate of isolates displaying non-wild-type (WT) phenotype, terbinafine minimum inhibitory concentrations (mg/L) and amino acid positions with hot-spot mutations in the squalene epoxidase target gene (where available) are presented.

The molecular mechanism underlying the reduced susceptibility to terbinafine has been correlated with hot-spot mutations in the SQLE target gene, which modify the protein structure leading to interference in drug’s binding to the target enzymes, mainly at the amino acid positions Leu393, Phe397, Phe415 and His440, with the first two being predominant (Figure 4, Table 4). Indeed, all terbinafine-resistant isolates found in the present study harbored the SQLE mutations Leu393 and Phe397 at similar frequencies (55% versus 45%, respectively). Nevertheless, several terbinafine-resistant *T. mentagrophytes* ITS Type VIII isolates with a non-mutated SQLE have been described [47,62,68,69] suggesting alternative mechanisms may confer resistance in clinical settings [70,71].

While the epidemic-like situation has begun as an India-centric phenomenon, the number of Asian and European countries witnessing infections due to terbinafine-resistant *T. mentagrophytes* ITS Type VIII isolates is rising steadily posing their transmission on the grounds of globalization as a serious issue to be considered even from a public health perspective. Figure 4 and Table 4 summarize all published studies where resistance in terbinafine was found for *T. interdigitale*/*T. mentagrophytes* clinical isolates. A switch to azole-based treatment may be necessary to cure such cases. In fact, patients presented in case reports/series (where data are available) responded well to oral (itraconazole, voriconazole) and/or simple topical azole (luliconazole, miconazole, eberconazole) therapy achieving a good therapeutic response [11,12,26,69,72,73,74]. Nonetheless, there are studies revealing a significant rate (up to 25%) of *T. mentagrophytes* Type VIII isolates with simultaneous in vitro resistance to both terbinafine and itraconazole [6,10,12]. Actually, the occurrence of cross-resistance between terbinafine and azoles is not surprising since both act on the ergosterol biosynthetic pathway by inhibiting the SQLE and the cytochrome P450-dependent lanosterol 14-α-demethylase, respectively. It is only recently that the role of overexpression of ATP-binding cassette transporter genes has been described as a mechanism of azole resistance in *T. mentagrophytes* [70].

Remarkably, in the present study, a considerably high rate (37.5%) of terbinafine non-WT *T. mentagrophytes* isolates was observed incorporating Greece in the increasingly expanding map of countries where antifungal resistance in dermatophytes has been documented. Based on the phylogenetic analysis by ITS dendrogram all the aforementioned formed their own clade belonging to Genotype VIII and were obtained predominately (87%) from cases of tinea cruris, which is in accordance with previous findings [21]. Sequence analysis of their SQLE-encoding gene demonstrated missense mutations leading to substituted amino acids in the SQLE protein. Strains with a Leu393Ser alteration showed lower terbinafine MICs compared to those possessing a Phe397Leu substitution (0.25–2 versus 2–8 mg/L, respectively), as previously described [6]. The amino acid substitution Ala448Thr at the C-terminus of the SQLE that has been associated with reduced susceptibility to azoles was not detected [6]. Indeed, all isolates retained susceptibility to itraconazole (MICs 0.016–0.125 mg/L) and voriconazole (MICs 0.03–0.5 mg/L), similarly to previous studies [24,64,72,73,74], and thus, could be considered as potential treatment candidates. In fact, a patient reported improvement after use of ointments of azoles combined with systemic itraconazole therapy (clinical metadata were missing in the rest of the cases). Notably, although the reason for the alarming upward trend in the incidence of recalcitrant dermatophytosis in India remains unclear, it has been suggested that the widespread availability and rampant use of over-the-counter topical preparations containing high-potency steroids and antifungals/antibacterials may have contributed to the acquisition of resistance [81,82]. Taking into account the case of *T. rubrum*, despite the fact that it appears to have limited capacity to develop resistance to terbinafine after prolonged drug exposure [83], long-term exposure to sub-inhibitory drug concentrations may favor the selection of resistant strains [84]. Nevertheless, in the present study, five out of seven patients were not pretreated with terbinafine indicating primary rather than acquired resistance. While it cannot be ruled out that some of them may have self-treated before arriving at the hospital, previous reports regarding the recovery of terbinafine-resistant *T. mentagrophytes* isolates from terbinafine-naive patients [10,33,69] and asymptomatic animals [78] corroborate our finding. Thus, the recovery of *T. mentagrophytes* Type VIII isolates in our country combined with high resistance to terbinafine underscores the need for reliable antifungal susceptibility testing particularly in non-responding dermatophytoses.

In vitro susceptibility testing of dermatophytes may be a key component of patient management, especially in cases of treatment failure and whenever prolonged therapy is required. Furthermore, it may help to distinguish between relapse (reinfection by the same pathogen) and reinfection (by a new pathogen), while it can assist in monitoring the epidemiological drug resistance patterns in a given region and thus defining local standard treatment guidelines. Although there are some indications of in vivo/in vitro discordance since laboratory data do not consistently predict a clinical response to terbinafine [85], it was reported that *T. interdigitale*/*T. mentagrophytes* isolates with Clinical and Laboratory Standards Institute (CLSI) MIC < 1 mg/L were associated with 2.5 higher cure rates than isolates with MICs ≥ 1 mg/L [62]. Nevertheless, it is important to highlight that there is currently no consensus on the optimal testing conditions and CLSI MIC data were generated using various modifications of the recommended reference methodology concerning the incubation length and temperature, the inoculum type (fragmented mycelial, conidial) and concentration and the reading MIC endpoint [13]. Indeed, highly variable terbinafine MIC ranges (MIC_50_) have been previously published for clinical *T. interdigitale*/*T. mentagrophytes* from 0.004–0.125 (0.016) to 0.25–≥32 (1) mg/L) [68] and for *T. rubrum* from 0.001–0.016 (0.008) to 0.03–0.5 (0.125) mg/L) [56,86] isolates, which may be attributed to inter-laboratory and inter-method variations suggesting a need for standardization and rendering comparison with our results difficult.

On the other hand, the recently released EUCAST reference method for antifungal susceptibility testing against microconidia-forming dermatophytes has been validated in a multicenter setting ensuring the reproducibility of MIC data [14]. This is the first study presenting MIC distributions of commonly used antifungals against *Trichophyton* spp. that were determined following the EUCAST E.DEF 11.0 and thus our data can only be accurately compared with those of the multicenter study [87]. For *T. rubrum* isolates, the mode MIC value of amorolfine in our study was identical to the spectrophotometric (spec)-50% MICs reported, while the modal MICs of voriconazole differed by one two-fold dilution (0.06 versus 0.03 mg/L). However, the mode MIC values of terbinafine and itraconazole were two two-fold dilutions higher (0.03 versus ≤0.008 mg/L and 0.06/0.125–0.016/≤ 0.03 mg/L, respectively). Being a tertiary care center, most patients presenting to us might have prior exposure to antifungals, antibiotics and/or steroids and this could be responsible for the higher MICs to some antifungals given that the MIC of quality control strains were at the target MIC. Moreover, heavy trailing growth observed for our isolates may have interfered with endpoint reading since differences were eliminated to one two-fold dilution for all antifungals when their spec-90% MICs were analyzed (0.06 versus 0.03 mg/L for terbinafine, 0.25 versus 0.125 mg/L for voriconazole, 0.5 versus 0.25 mg/L for itraconazole and 0.06 versus 0.125 mg/L for amorolfine). Despite the highly variable terbinafine CLSI MIC ranges mentioned before, overall most strains display CLSI terbinafine MIC_50_ values of 0.03–0.06 mg/L, which we could regard as equivalent to our findings [8,45,54,57,88,89]. Hence, a larger-scale study of global isolates is warranted to indicate whether a more stringent EUCAST spec reading endpoint is preferable for *T. rubrum*. Regarding our *T. interdigitale* isolates, the modal MICs of all antifungals differed from the previously reported by one or two two-fold dilutions (≤0.008 versus 0.03 mg/L for terbinafine, 0.06 versus 0.125 mg/L for voriconazole, 0.03 versus 0.06 mg/L for itraconazole and 0.06 versus 0.125 mg/L for amorolfine), as opposed to the mode MIC values of our *T. mentagrophytes* strains that were almost identical [87]. Of note, the 20 *T. interdigitale* isolates used in the multicenter validation study of the EUCAST methodology were of Indian origin (personal communication with Prof. M.C. Arendrup) [10] and were all retrospectively identified by phylogenetic analysis as *T. mentagrophytes* ITS Type VIII based on the new taxonomy of dermatophytes [64]. In our view, the aforementioned finding emphasizes the need for further studies involving isolates with geographical and genotypic diversity in order to define the formal ECOFFs as the inclusion of such isolates will enable the analysis of genotype-related differences in MIC distributions, if any.

Taken together, our study aids in understanding the local genotypic and antifungal susceptibility patterns of *Trichophyton* spp. While terbinafine does not warrant a reappraisal of its utility as a front-line drug for the treatment of *T. rubrum* dermatophytosis, the current high recovery rate of terbinafine non-WT *T. mentagrophytes* ITS Type VIII strains from Greek residents raises concerns. Overall, there is a need for surveillance studies to establish baseline in vitro susceptibility data that will allow monitoring the antifungal resistance trends in dermatophytes and defining ECOFFs with ultimate goal the optimization of antifungal therapy by minimizing the administration of inappropriate chosen drugs and experimentation with doses and frequency.

## Figures and Tables

**Table 1:** In vitro susceptibility profile of 112 Greek *Trichophyton* spp. clinical isolates determined with the EUCAST E.DEF 11.0 [14].

Species (No of Isolates)	Antifungal Agent		Number of Isolates with MIC (mg/L) of:										MIC_50_/MIC_90_	GM MIC	% Non-WT Phenotype
		≤0.008	0.016	0.03	0.06	0.125	0.25	0.5	1	2	4	8			
*T. rubrum* (*n* = 70)	TRB	2	29	39	-	-	-	-	-	-	-	-	0.03/0.03	0.022	0%
	VRC	-	3	16	31	20	-	-	-	-	-	-	0.06/0.125	0.060	0%
	ITC	-	1	20	21	21	7	-	-	-	-	-	0.06/0.25	0.069	0%
	AMO	-	7	24	31	8	-	-	-	-	-	-	0.06/0.125	0.045	0%
															
*T. interdigitale* (*n* = 12)	TRB	6	3	3	-	-	-	-	-	-	-	-	≤0.008/0.03	0.013	0%
	VRC	-	2	3	5	2	-	-	-	-	-	-	0.06/0.125	0.046	0%
	ITC	1	1	6	4	-	-	-	-	-	-	-	0.03/0.06	0.032	0%
	AMO	-	-	3	5	4	-	-	-	-	-	-	0.06/0.125	0.064	0%
															
*T. mentagrophytes* (*n* = 24)	TRB	1	4	7	3	-	2 ^a^	-	-	4 ^a^	2 ^a^	1 ^a^	0.03/4	0.127	37.5% ^b^
	VRC	-	1	1	6	9	4	3	-	-	-	-	0.125/0.5	0.120	0% ^b^
	ITC	-	2	5	7	9	1	-	-	-	-	-	0.06/0.125	0.065	0% ^b^
	AMO	-	-	1	1	8	13	1	-	-	-	-	0.25/0.25	0.176	0% ^b^
															
*T. tonsurans* (*n* = 6)	TRB	-	6	-	-	-	-	-	-	-	-	-	0.016/0.016	0.016	0% ^b^
	VRC	-	-	1	2	1	2	-	-	-	-	-	0.06/0.25	0.097	0% ^b^
	ITC	-	-	3	3	-	-	-	-	-	-	-	0.03/0.06	0.042	0% ^b^
	AMO	-	3	1	-	2	-	-	-	-	-	-	0.016/0.125	0.035	0% ^b^

Non-WT isolates are shaded and modal MICs are indicated with underlined numbers. ^a^: *T. mentagrophytes* ITS Type VIII isolates. ^b^: The tentative epidemiological cut-off values of *T. interdigitale* were used. Abbreviations: MIC: minimum inhibitory concentration, GM: geometric mean, WT: wild-type, TRB: terbinafine, ITC: itraconazole, VRC: voriconazole, AMO: amorolfine.

**Table 2:** Overview of the patients with dermatophytosis due to *T. mentagrophytes* ITS Type VIII exhibiting reduced susceptibility to terbinafine.

Isolate	Gender/ Age (Years)	Tinea Infection	Nationality; Additional Remarks	Sampling Date	Antifungals MICs (mg/L); Interpretation				Amino Acid Substitution within the SQLE (Codon Change)
					TRB	VRC	ITC	AMO	
AUH1273	M/67	Tinea cruris	Greek Prior use of ointments of azoles	05/2018	8	0.06 WT	0.03 WT	0.25 WT	Phe397Leu (TTC→TTA)
AUH1357	F/42	Tinea cruris	Greek Resident of a Roma camp Prior use of ointments of azoles and systemic/topical treatment with TRB	08/2018	2	0.06 WT	0.03 WT	0.25 WT	Leu393Ser (TTA→TCA)
AUH1665	F/42 (Same as AUH1357)	Tinea cruris and tinea corporis	No improvement after use of ointments of azoles and systemic treatment with TRB	13/11/2019	2	0.06 WT	0.03 WT	0.25 WT	Leu393Ser (TTA→TCA)
AUH1678	F/42 (Same as AUH1357)			28/11/2019	2	0.125 WT	0.06 WT	0.25 WT	Leu393Ser (TTA→TCA)
AUH1598	M/33	Tinea cruris	Iranian Prior use of ointments of azoles	08/2019	0.25	0.25 WT	0.125 WT	0.125 WT	Leu393Ser (TTA→TCA)
AUH1621	M/69	Tinea cruris and tinea corporis	Greek No prior use of antifungals—improvement after use of ointments of azoles and systemic treatment with ITC	10/2019	2	0.125 WT	0.06 WT	0.125 WT	Phe397Leu (TTC→TTA)
AUH1650	M/0.8	Tinea cruris	Syrian Resident of a refugee camp Prior use of ointments of azoles	10/2019	0.25	0.5 WT	0.125 WT	0.25 WT	Leu393Ser (TTA→TCA)
AUH1687	M/24	Tinea cruris	Greek Prior use of ointments of azoles and systemic treatment with TRB	11/2019	4	0.03 WT	0.016 WT	0.125 WT	Phe397Leu (TTC→TTA)
AUH1745	F/90	Tinea corporis	Greek Prior systemic treatment with FLC	12/2019	4	0.5 WT	0.06 WT	0.25 WT	Phe397Leu (TTC→TTA)

Abbreviations: M: male, F: female, MIC: minimum inhibitory concentration, WT: wild-type, TRB: terbinafine, ITC: itraconazole, VRC: voriconazole, AMO: amorolfine, FLC: fluconazole, SQLE: squalene epoxidase.

**Table 3:** Globally reported *T. rubrum* clinical isolates exhibiting reduced susceptibility to terbinafine.

Country	Type of Study	Sampling Year	Tinea Infection	No of Isolates	AST Method (Protocol)	TRB MIC (mg/L); % of Non-WT Isolates	Other Antifungals Tested against TRB Non-WT Isolates (MIC (mg/L), % of Non-WT Isolates)	Amino Acid Substitution within the SQLE (Codon Change)
Denmark [38]	Case report	ND	Tinea corporis, tinea pedis	1	BMD (EUCAST E.Def 9.3)	4; 100%	FLC (4, 0%) ITC (0.125, 0%) ISA (0.125, 0%) VRC (0.125, 0%) POS (0.03, 0%)	Phe397Leu [7] (NA)
Denmark [39]	Case report	ND	Tinea corporis	1	BMD (EUCAST E.Def 9.3)	>4; 100%	ITC (0.03, 0%)	Phe397Leu [7] (NA)
Denmark [7]	Case series	ND	Various typesof tinea	10	BMD (EUCAST E.Def 11.0)	0.125–>8; 100%	ND	Phe397Leu (*n* = 4) Leu393Ser (*n* = 2) ^b^ Leu393Phe (*n* = 1) Phe415Ser (*n* = 1) ^c^ His440Tyr, Phe484Tyr (*n* = 1) ^c^ Ile121Met, Val237Ile (*n* = 1) ^c^ (NA)
India [6]	Multicenter laboratory-based (screening of *Trichophyton* spp. clinical isolates)	2017–2019	Various typesof tinea (mainly tinea corporis and tinea cruris)	18	Agar screening(SDA containing TRB 0.2 mg/L) BMD (CLSI M38-A2)	0.03–8; 44%	NA ^d^	Phe397Leu (TTC→CTC)
India [44]	Single-center laboratory-based (screening of *Trichophyton* spp. clinical isolates recovered from recalcitrant/refractory cases)	2014–2017	Various typesof tinea (mainly tinea corporis and tinea cruris)	13	BMD (CLSI M38-A2)	0.125–8; 54%	NA ^d^	ND
India [45]	Single-center laboratory-based (screening of *Trichophyton* spp. clinical isolates recovered from recurrent cases)	2015	Various typesof tinea (mainly tinea corporis and tinea cruris)	29	BMD (CLSI M38-A2)	0.016–16; 10%	NA ^d^	ND
India [46]	Single-center laboratory-based (screening of *Trichophyton* spp. clinical isolates)	2014–2015	Tinea corporis, tinea cruris	5	BMD (CLSI M38-A2)	0.03–8; 40%	NA ^d^	ND
India [47]	Single-center laboratory-based (screening of *Trichophyton* spp. clinical isolates)	2014	Various typesof tinea (mainly tinea corporis and tinea cruris)	35	BMD (CLSI M38-A2)	0.016–16; 14%	NA ^d^	Phe397Leu (*n* = 1) (TTC→CTC) WT (*n* = 3) (-)
India [31]	Multicenter laboratory-based (screening of *Trichophyton* spp. clinical isolates) [48]	ND	Various typesof tinea (mainly tinea corporis and tinea cruris)	18	BMD (CLSI M38-A2)	0.03–4; 11%	NA^d^	ND
Iran [8]	Multicenter laboratory-based (screening of *Trichophyton* spp. clinical isolates)	ND	Tinea corporis, tinea pedis	20	BMD (CLSI M38-A2)	0.004–> 32; 10%	LLC (NA, 0%) ^d^	Leu393Phe (*n* = 1) (TTA→TTT) WT (*n* = 1) (-)
Japan [32]	Multicenter laboratory-based (screening of *Trichophyton* spp. clinical isolates)	2020	Various typesof tinea (mainly tinea pedis and tinea corporis)	128	Agar screening(SDA containing TRB 1 mg/L) BMD (CLSI M38-A2)	32–>32; 4%	ITC (≤0.03–0.25, 0%) RVC (≤0.03–0.06, 0%) LLC (≤0.03, 0%)	Leu393Phe (1179A→C/T)
Japan [37]	Case report	2017 2019	Tinea unguium (fingernails)	2 ^a^	BMD (CLSI M38-A2)	8–16; 100%	ITC (0.06–0.25, 0%) RVC (≤0.03, 0%)	Phe397Leu (TTC→TTA)
Japan [40]	Case report	2016	Tinea pedis	1	BMD (CLSI M38-A2)	>128; 100%	ITC (0.03, 0%)	Leu393Phe (TTA→TTC)
Malaysia [41]	Single-center laboratory-based (screening of *Trichophyton* spp. clinical isolates)	2012–2013	Various types of tinea	3	BMD (CLSI M38-A2)	2–4; 100%	AMB (0.06–0.125, 0%) ITC (0.5, 0%) CLT (0.06–0.125, 0%) KTC (0.25–0.5, 0%) MCZ (0.5, 0%)	ND
United States of America [36]	Case report	ND	Tinea corporis, tinea unguium (toenails)	1	BMD (CLSI M38-A2)	>0.5; 100%	FLC (≤0.03, 0%)	ND
North America [43]	Case report	ND [49]	*Tinea* unguium (toenails)	6 ^a^	BMD (CLSI M27-A)	4; 100%	FLC (0.25–0.5, 0%) ITC (≤0.06, 0%) GRS (0.125–0.5, 0%)	Leu393Phe [50] (TTA→TTC)
Switzerland [9]	Multicenter laboratory-based (screening of *Trichophyton* spp. clinical isolates)	2013–2016	Tinea unguium, tinea pedis	1644	Agar screening(SDA containing TRB 0.2 mg/L) BMD only for TRB non-WT isolates (CLSI M38-A2)	1–>128; 1%	ND	Leu393Phe (*n* = 4) (TTA→TTT) Leu393Ser (*n* = 2) (TTA→TCA) Phe397Leu (*n* = 4) (TTC→TTA/TTC→CTC) Phe397Ile (*n* = 1) (TTC→ATC) Phe397Val (*n* = 1) (TTC→GTC) Phe415Ile (*n* = 1) ^b^ (TTC→ATC) Phe415Ser (*n* = 1) (TTC→TCC) Phe415Val (*n* = 1) (TTC→GTC) His440Tyr (*n* = 1) ^b^ (CAT→TAT)
Switzerland [42]	Case report	ND	ND	1	BMD (CLSI M38-A)	64; 100%	FLC (NA, 0%) ITC (NA, 0%) GRS (NA, 0%)	Phe397Leu (TTC→TTA)

^a^: obtained sequentially from a single patient; ^b^: moderately-resistant isolates (TRB MIC 1 mg/L); ^c^: low-resistant isolates (TRB MIC 0.125–0.25 mg/L); ^d^: several antifungals have been tested, but isolates with a non-WT phenotype to TRB have not been analyzed separately. Abbreviations: NA: not available, ND: not determined, TRB: terbinafine, FLC: fluconazole, ITC: itraconazole, ISA: isavuconazole, POS: posaconazole, VRC: voriconazole, RVC: ravuconazole, LLC: luliconazole, GRS: griseofulvin, KTC: ketoconazole, MCZ: miconazole, CLT: clotrimazole, AMB: Amphotericin B, MIC: minimum inhibitory concentration, AST: antifungal susceptibility testing, WT: wild-type, BMD: broth microdilution method, SDA: Sabouraud dextrose agar, SQLE: squalene epoxidase.

**Table 4:** Global distribution of *T. interdigitale*/*T. mentagrophytes* clinical isolates exhibiting reduced susceptibility to terbinafine.

Country	Type of Study	Sampling Year	Tinea Infection	No of Isolates (Species)	AST Method (Protocol)	TRB MIC (mg/L); % of Non-WT Isolates	Other Antifungals Tested against TRB Non-WT Isolates (MIC (mg/L), % of Non-WT Isolates)	Amino Acid Substitution within the SQLE (Codon Change)
Belgium [75]	Multicenter laboratory-based (screening of *Trichophyton* spp. clinical isolates)	2018	Tinea capitis	5 (*Tm*)	BMD (EUCAST E.Def 11.0)	0.016–4; 20%	ITC (0.016, 0%) VRC (0.5, 0%) AMO (0.06, 0%)	Phe397Leu (NA)
Denmark [7]	Case series	ND	Various typesof tinea	2 (*Ti*)	BMD (EUCAST E.Def 11.0)	>4–>8; 100%	ND	Phe397Leu (*n* = 1) Leu393Phe (*n* = 1) (NA)
Finland [76]	Case series	2019	Tinea cutis glabrae	4 (*Tm* VIII)	BMD (ND)	4–>8 (data available only for non-WT isolates); 75%	ND	ND
Germany [12]	Case series	2016–2020 (72% during 2019–2020)	Various types of tinea (mainly tinea corporis and tinea cruris)	29 (*Tm* VIII)	Agar screening (SDA containing TRB 0.2 mg/L) BMD (CLSI M38-A2)	<0.2–16; 45%	ITC (0.008–0.5, 20%) ^a^ VRC (0.008–0.25, 20%) ^a^	Phe397Leu (*n* = 10) (TTC→CTC/TTC→TTA) Phe397Leu, Ala448Thr (*n* = 2) (TTC→CTC, GCT→ACT) Leu393Phe (*n* = 1) (TTA→TTC)
Germany [77]	Case report	2019	Tinea corporis, tinea cruris	1 (*Tm* VIII)	ND	ND	ND	Phe397Leu (TTC→CTC)
Germany [26]	Case report	ND	Tinea corporis	1 (*Tm* VIII)	Agar screening(SDA containing TRB 0.2 mg/L)	ND	ND	Phe397Leu (TTC→TTA)
Greece (present study)	Single-center laboratory-based (screening of *Trichophyton* spp. clinical isolates)	2010–2019	Various types of tinea	24 (*Tm*)	BMD (EUCAST E.Def 11.0)	0.008–8; 37.5% (all *Tm* VIII)	ITC (0.016–0.125, 0%) VRC (0.03–0.5, 0%) AMO (0.125–0.5, 0%)	Leu393Ser (*n* = 5) (TTA→TCA) Phe397Leu (*n* = 4) (TTC→TTA)
India [6]	Multicenter laboratory-based (screening of *Trichophyton* spp. clinical isolates)	2017–2019	Various types of tinea (mainly tinea corporis and tinea cruris)	279 (*Tm* VIII)	Agar screening(SDA containing TRB 0.2 mg/L) BMD (CLSI M38-A2)	0.125–16; 71%	NA ^d^	Phe397Leu (*n* = 153) (1189T→C/1191C→A/G) Phe397Leu, Ala448Thr (*n* = 27) (1189T→C/1191C→A/G, 1342G→A) Leu393Ser (*n* = 7) ^b^ (1178T→C) Leu393Phe (*n* = 6) (1179A→C) His440Tyr (*n* = 2) ^c^ (1318C→T) Gln408Leu, Ala448Thr (*n* = 2) ^b^ (1223A→T, 1342G→A) Ser443Pro (*n* = 1) ^c^ (1327T→C) Ser395Pro, Ala448Thr (*n* = 1) ^c^ (1183T→C, 1342G→A)
India [68]	Multicenter laboratory-based (screening of *Ti/Tm* clinical isolates)	2014–2018	Various types of tinea	498 (*Ti/Tm*)	BMD (CLSI M38-A2)	0.016–32; 11%	NA ^d^	Phe397Leu (*n* = 43) (NA) WT (*n* = 14) (-)
India [64]	Multicenter laboratory-based (screening of *Trichophyton* spp. clinical isolates)	2014–2018	Various types of tinea (mainly tinea corporis and tinea cruris)	129 (*Ti/Tm*)	BMD (CLSI M38-A2)	0.125–32; 37%	ITC (0.06–2, 2%) VRC (0.03–0.5, 0%) FLC (0.5–64, 20%) LLC (0.004–0.03, 0%) CLT (1–8, 78%) MCZ (0.5–4, 80%) KTC (0.25–8, 9%) GRS (2–>8, 100%) STC (0.125- > 16, 35%)	Phe397Leu (*n* = 39) Leu393Phe (*n* = 7) (NA)
India [33]	Single-center laboratory-based (screening of *Trichophyton* spp. clinical isolates)	2017	Various types of tinea (mainly tinea corporis and tinea cruris)	ND (97 patients with *Tm* infection)	BMD (CLSI M38-A2)	2–16 (data available only for non-WT isolates); 15 isolates obtained from 13 patients (13%)	ND	Phe397Leu (TTC→CTC/TTC→TTA)
India [62]	Single-center laboratory-based (screening of *Trichophyton* spp. clinical isolates)	2016–2017	Tinea corporis, tinea cruris	64 (*Ti*)	BMD (CLSI M38-A2)	0.25–>32; 61%	NA ^d^	Phe397Leu (*n* = 10) ^a^ Leu393Phe (*n* = 3) WT (*n* = 4) (NA)
India [10]	Multicenter laboratory-based (screening of *Trichophyton* spp. clinical isolates)	2015–2017	Various types of tinea (mainly tinea corporis and tinea cruris)	63 (*Ti*)	BMD (CLSI M38-A2)	0.06–>32; 32%	ITC (0.06–> 16, 25%) VRC (0.06–> 16, 10%) FLC (0.5–> 64, 80%) LLC (≤ 0.004–0.5, 0%) CLT (2–16, 100%) MCZ (1–> 16, 80%) KTC (0.5–> 32, 35%) GRS (2–> 8, 100%) AMB (0.25–1, 0%) STC (0.5–> 16, 75%)	Phe397Leu (*n* = 12) Leu393Phe (*n* = 8) (NA)
India [44]	Single-center laboratory-based (screening of *Trichophyton* spp. clinical isolates recovered from recalcitrant/refractory cases)	2014–2017	Various types of tinea (mainly tinea corporis and tinea cruris)	31 (*Tm*)	BMD (CLSI M38-A2)	0.03–16; 74%	NA ^d^	ND
India [45]	Single-center laboratory-based (screening of *Trichophyton* spp. clinical isolates recovered from recurrent cases)	2015	Various types of tinea (mainly tinea corporis and tinea cruris)	36 (*Tm*) 10 (*Ti*)	BMD(CLSI M38-A2)	*Tm*: 0.016–8; 11% *Ti*: 0.016–8; 10%	NA ^d^	ND
India [46]	Single-center laboratory-based (screening of *Trichophyton* spp. clinical isolates)	2014–2015	Various types of tinea (mainly tinea corporis and tinea cruris)	37 (*Ti*)	BMD (CLSI M38-A2)	0.03–16; 8%	NA ^d^	ND
India [47]	Single-center laboratory-based (screening of *Trichophyton* spp. clinical isolates)	2014	Various types of tinea (mainly tinea corporis and tinea cruris)	88 (*Ti*)	BMD (CLSI M38-A2)	0.016–32; 17%	NA ^d^	Phe397Leu (*n* = 4) (TTC→CTC) WT (*n* = 11) (-)
India [31]	Multicenter laboratory-based (screening of *Trichophyton* spp. clinical isolates) [48]	ND	Various types of tinea (mainly tinea corporis and tinea cruris)	34 (*Tm*)	BMD (CLSI M38-A2)	0.06–4; 24%	NA ^d^	ND
Iran [65]	Multicenter laboratory-based (screening of *Ti/Tm* clinical isolates)	2016–2018	Various types of tinea	45 (28 *Tm* VIII, 17 *Tm*)	BMD (CLSI M38-A2)	0.008–>32; 11% (all *Tm* VIII)	ITC (0.125–2, NA) LLC (0.004–0.008, 0%) GRS (1–4, NA) EFC (0.002–0.008, 0%) CLT (1–8, NA) AMO (0.5–2, NA)	Phe397Leu, Ala448Thr (*n* = 4) Leu393Ser, Ala448Thr (*n* = 4) (NA)
Iran [21]	Multicenter laboratory-based (screening of *Ti/Tm* clinical isolates)	2016–2018	Various types of tinea	140 (45 *Tm*, 95 *Tm*)	BMD (CLSI M38-A2)	*Tm*: 0.004–>32; 11% (all *Tm* VIII) *Ti*: 0.004–0.25; 0%	ND	ND
Iran [11]	Case series	ND	Various types of tinea	4 (*Tm* VIII)	BMD (CLSI M38-A2)	>8; 100%	ITC (≥ 4, 100%) FLC (≥ 16, 100%) VRC (0.25–0.5, 0%) POS (0.03–0.06, 0%)	Phe397Leu (TTC→TTA)
Japan [69]	Case report	2017–2018	Tinea pedis	1 (*Ti*)	BMD for TRB (CLSI M38-A) E-test for ITC	2; 100%	ITC (0.5, 0%)	WT (-)
Japan [73]	Case report	ND	Tinea corporis	1 (*Ti*)	BMD (CLSI M38-A2)	32; 100%	ITC (≤ 0.03, 0%) RVC (≤ 0.03, 0%)	Phe397Leu (NA)
Japan [72]	Case report	ND	Tinea corporis, tinea cruris, tinea faciei	1 (*Ti*)	BMD (CLSI M38-A2)	>32; 100%	ITC (0.03, 0%) RVC (0.5, 0%) LLC (≤ 0.03, 0%) CLT (4, 100%) MCZ (8, 100%)	Phe397Leu (NA)
Poland [78]	Multicenter laboratory-based (screening of *Tm* isolates)	2016–2019	Tinea capitis, tinea unguium	7 (*Tm*)	BMD (CLSI M38-A2)	0.004–32; 14%	NA ^d^	Leu393Phe (NA)
Russian Federation [79]	Multicenter laboratory-based (screening of *Tm* isolates)	2015–2018	Strains isolated from symptomatic animals (cats and dogs)	10 (*Tm*)	BMD (EUCAST E.Def 9.3.1)	>32 (data available only for non-WT isolates); 40%	ENC (NA, 0%) KTC (NA, 0%)	ND
Switzerland [24]	Multicenter laboratory-based (screening of *Ti/Tm* clinical isolates)	2009–2019	Various types of tinea (mainly tinea corporis and tinea faciei)	162 (*Tm*)	BMD [80]	>4 (data available only for non-WT isolates); 2% (all *Tm* VIII)	ITC (NA, 0%) FLC (NA, 0%) KTC (NA, 0%) GRS (NA, 0%)	Phe397Leu (NA)
Switzerland [9]	Multicenter laboratory-based (screening of *Trichophyton* spp. clinical isolates)	2013–2016	Tinea unguium	412 (*Ti*)	Agar screening(SDA containing TRB 0.2 mg/L) BMD only for TRB non-WT isolates (CLSI M38-A2)	32; 0.2%	ND	Phe397Leu (TTC→CTC)
Switzerland [80]	Single-center laboratory-based (screening of *Trichophyton* spp. clinical isolates)	ND	ND	7 (*Tm*)	BMD [80]	≤0.004–>8; 14%	NA ^d^	Phe397Leu(NA)
Switzerland [74]	Case report	ND	Tinea corporis	2 (*Tm*)	BMD for TRB (CLSI M38-A) Sensititre YeastOne for azoles	>1; 100%	ITC (0.016, 0%) POS (0.008, 0%)	Gln408Leu (CAA→CTA)

^a^: data available for a proportion of isolates; ^b^: moderately-resistant isolates (TRB MIC 1 mg/L); ^c^: low-resistant isolates (TRB MIC 0.125–0.25 mg/L); ^d^: several antifungals have been tested, but isolates with a non-WT phenotype to TRB have not been analyzed separately. Abbreviations: NA: not available, ND: not determined, *Ti*: *T. interdigitale*, *Tm*: *T. mentagrophytes*, *Tm* VIII: *T. mentagrophytes* Type VIII (India), TRB: terbinafine, FLC: fluconazole, ITC: itraconazole, POS: posaconazole, VRC: voriconazole, RVC: ravuconazole, LLC: luliconazole, GRS: griseofulvin, KTC: ketoconazole, MCZ: miconazole, CLT: clotrimazole, AMB: Amphotericin B, AMO: amorolfine, STC: sertaconazole, ENC: enilconazole, EFC: efinaconazole, MIC: minimum inhibitory concentration, AST: antifungal susceptibility testing, WT: wild-type, BMD: broth microdilution method, SDA: Sabouraud dextrose agar, SQLE: squalene epoxidase.

## Data Availability

Data available on request.

## References

[1] Havlickova B., Czaika V.A., Friedrich M. (2008). Epidemiological trends in skin mycoses worldwide. Mycoses.

[2] Benedict K., Jackson B.R., Chiller T., Beer K.D. (2019). Estimation of Direct Healthcare Costs of Fungal Diseases in the United States. Clin. Infect. Dis..

[3] Fiammenghi E., Patalano A., Conte V.L., Calabrò G. (2015). Cost analysis of inappropriate treatments for suspected dermatomycoses. Farmeconomia Health Econ. Ther. Pathw..

[4] Monod M. (2019). Antifungal resistance in dermatophytes: Emerging problem and challenge for the medical community. J. Med. Mycol..

[5] Hay R. (2018). Therapy of Skin, Hair and Nail Fungal Infections. J. Fungi.

[6] Ebert A., Monod M., Salamin K., Burmester A., Uhrlaß S., Wiegand C., Hipler U., Krüger C., Koch D., Wittig F. (2020). Alarming India-wide phenomenon of antifungal resistance in dermatophytes: A multicentre study. Mycoses.

[7] Saunte D.M.L., Hare R.K., Jørgensen K.M., Jørgensen R., Deleuran M., Zachariae C.O., Thomsen S.F., Bjørnskov-Halkier L., Kofoed K., Arendrup M.C. (2019). Emerging Terbinafine Resistance in Trichophyton: Clinical Characteristics, Squalene Epoxidase Gene Mutations, and a Reliable EUCAST Method for Detection. Antimicrob. Agents Chemother..

[8] Salehi Z., Shams-Ghahfarokhi M., Razzaghi-Abyaneh M. (2018). Antifungal drug susceptibility profile of clinically important dermatophytes and determination of point mutations in terbinafine-resistant isolates. Eur. J. Clin. Microbiol. Infect. Dis..

[9] Yamada T., Maeda M., Alshahni M.M., Tanaka R., Yaguchi T., Bontems O., Salamin K., Fratti M., Monod M. (2017). Terbinafine Resistance of *Trichophyton* Clinical Isolates Caused by Specific Point Mutations in the Squalene Epoxidase Gene. Antimicrob. Agents Chemother..

[10] Singh A., Masih A., Khurana A., Singh P.K., Gupta M., Hagen F., Meis J.F., Chowdhary A. (2018). High terbinafine resistance in *Trichophyton interdigitale* isolates in Delhi, India harbouring mutations in the squalene epoxidase gene. Mycoses.

[11] Fattahi A., Shirvani F., Ayatollahi A., Rezaei-Matehkolaei A., Badali H., Lotfali E., Ghasemi R., Pourpak Z., Firooz A. (2020). Multi-drug-resistant *Trichophyton mentagrophytes* genotype VIII in an Iranian family with generalized dermatophytosis: Report of four cases and review of literature. Int. J. Dermatol..

[12] Nenoff P., Verma S.B., Ebert A., Süß A., Fischer E., Auerswald E., Dessoi S., Hofmann W., Schmidt S., Neubert K. (2020). Spread of Terbinafine-Resistant *Trichophyton mentagrophytes* Type VIII (India) in Germany–“The Tip of the Iceberg?”. J. Fungi.

[13] Rudramurthy S.M., Dogra S., Shaw D. (2019). Antifungal drug susceptibility testing of dermatophytes: Laboratory findings to clinical implications. Indian Dermatol. Online J..

[14] Arendrup M.C., Kahlmeter G., Guinea J., Meletiadis J., Arikan-Akdagli S., Friberg N., Barchiesi F., Castanheira M., Hamal P., Järv H. (2021). How to: Perform antifungal susceptibility testing of microconidia-forming dermatophytes following the new reference EUCAST method E.Def 11.0, exemplified by *Trichophyton*. Clin. Microbiol. Infect..

[15] De Hoog G.S., Gene J., Ahmed S., Al-Hatmi A.M.S., Figueras M.J., Vitale R.G. (2001). Atlas of Clinical Fungi.

[16] Alastruey-Izquierdo A., Mellado E., Peláez T., Pemán J., Zapico S., Alvarez M., Rodríguez-Tudela J.L., Cuenca-Estrella M. (2013). FILPOP Study Group Population-Based Survey of Filamentous Fungi and Antifungal Resistance in Spain (FILPOP Study). Antimicrob. Agents Chemother..

[17] Habeb K.A., Maikhan H.K., Rachid S.K. (2016). Molecular Identification of Dermatophytes among Clinical Isolates. Asian J. Nat. Appl. Sci..

[18] Nenoff P., Verma S.B., Uhrlaß S., Burmester A., Gräser Y. (2018). A clarion call for preventing taxonomical errors of dermatophytes using the example of the novel *Trichophyton mentagrophytes* genotype VIII uniformly isolated in the Indian epidemic of superficial dermatophytosis. Mycoses.

[19] Lipner S.R., Scher R.K. (2019). Onychomycosis: Treatment and prevention of recurrence. J. Am. Acad. Dermatol..

[20] Kumar S., Stecher G., Li M., Knyaz C., Tamura K. (2018). MEGA X: Molecular evolutionary genetics analysis across computing platforms. Mol. Biol. Evol..

[21] Taghipour S., Pchelin I.M., Mahmoudabadi A.Z., Ansari S., Katiraee F., Rafiei A., Shokohi T., Abastabar M., Taraskina A.E., Kermani F. (2019). *Trichophyton mentagrophytes* and *T. interdigitale* genotypes are associated with particular geographic areas and clinical manifestations. Mycoses.

[22] Nenoff P., Verma S.B., Vasani R., Burmester A., Hipler U., Wittig F., Krüger C., Nenoff K., Wiegand C., Saraswat A. (2018). The current Indian epidemic of superficial dermatophytosis due to *Trichophyton mentagrophytes*—A molecular study. Mycoses.

[23] Heidemann S., Monod M., Gräser Y. (2009). Signature polymorphisms in the internal transcribed spacer region relevant for the differentiation of zoophilic and anthropophilic strains of *Trichophyton interdigitale* and other species of *T. mentagrophytes sensu lato*. Br. J. Dermatol..

[24] Klinger M., Theiler M., Bosshard P. (2021). Epidemiological and clinical aspects of *Trichophyton mentagrophytes/Trichophyton interdigitale* infections in the Zurich area: A retrospective study using genotyping. J. Eur. Acad. Dermatol. Venereol..

[25] Kupsch C., Czaika V., Deutsch C., Gräser Y. (2019). *Trichophyton mentagrophytes*—a new genotype of zoophilic dermatophyte causes sexually transmitted infections. J. Dtsch. Dermatol. Ges..

[26] Süß A., Uhrlaß S., Ludes A., Verma S.B., Monod M., Krüger C., Nenoff P. (2019). Ausgeprägte Tinea corporis durch ein Terbinafin-resistentes *Trichophyton-mentagrophytes*-Isolat vom indischen Genotyp bei einem Säugling aus Bahrain in Deutschland. Der Hautarzt.

[27] Amnesty International, Trapped in Greece: An Avoidable Refugee Crisis. https://www.refworld.org/docid/571db6df4.html.

[28] Dogra S., Kaul S., Yadav S. (2017). Treatment of dermatophytosis in elderly, children, and pregnant women. Indian Dermatol. Online J..

[29] Rouzaud C., Chosidow O., Brocard A., Fraitag S., Scemla A., Anglicheau D., Bouaziz J.-D., Dupin N., Bougnoux M.-E., Hay R. (2018). Severe dermatophytosis in solid organ transplant recipients: A French retrospective series and literature review. Transpl. Infect. Dis..

[30] Liu H.B., Liu F., Kong Q.T., Shen Y.N., Lv G.X., Liu W.D., Sang H. (2015). Successful Treatment of Refractory Majocchi’s Granuloma with Voriconazole and Review of Published Literature. Mycopathologia.

[31] Bhatia V., Sharma P. (2015). Determination of minimum inhibitory concentrations of itraconazole, terbinafine and ketoconazole against dermatophyte species by broth microdilution method. Indian J. Med Microbiol..

[32] Hiruma J., Noguchi H., Hase M., Tokuhisa Y., Shimizu T., Ogawa T., Hiruma M., Harada K., Kano R. (2021). Epidemiological study of terbinafine-resistant dermatophytes isolated from Japanese patients. J. Dermatol..

[33] Shankarnarayan S.A., Shaw D., Sharma A., Chakrabarti A., Dogra S., Kumaran M.S., Kaur H., Ghosh A., Rudramurthy S.M. (2020). Rapid detection of terbinafine resistance in *Trichophyton* species by Amplified refractory mutation system-polymerase chain reaction. Sci. Rep..

[34] Rezaei-Matehkolaei A., Khodavaisy S., Alshahni M.M., Tamura T., Satoh K., Abastabar M., Shokoohi G.R., Ahmadi B., Kord M., Taghipour S. (2018). In Vitro Antifungal Activity of Novel Triazole Efinaconazole and Five Comparators against Dermatophyte Isolates. Antimicrob. Agents Chemother..

[35] Ansari S., Hedayati M.T., Zomorodian K., Pakshir K., Badali H., Rafiei A., Ravandeh M., Seyedmousavi S. (2016). Molecular Characterization and In Vitro Antifungal Susceptibility of 316 Clinical Isolates of Dermatophytes in Iran. Mycopathologia.

[36] Gu D., Hatch M., Ghannoum M., Elewski B.E. (2020). Treatment-resistant dermatophytosis: A representative case highlighting an emerging public health threat. JAAD Case Rep..

[37] Noguchi H., Matsumoto T., Hiruma M., Kimura U., Kano R., Yaguchi T., Fukushima S., Ihn H. (2019). Tinea unguium caused by terbinafine-resistant *Trichophyton rubrum* successfully treated with fosravuconazole. J. Dermatol..

[38] Schøsler L., Andersen L.K., Arendrup M.C., Sommerlund M. (2018). Recurrent terbinafine resistant *Trichophyton rubrum* infection in a child with congenital ichthyosis. Pediatr. Dermatol..

[39] Wingfield Digby S.S., Hald M., Arendrup M.C., Hjorth S.V., Kofoed K. (2017). Darier disease complicated by terbinafine-resistant *Trichophyton rubrum*: A case report. Acta Derm. Venereol..

[40] Suzuki S., Mano Y., Furuya N., Fujitani K. (2018). Discovery of Terbinafine Low Susceptibility *Trichophyton rubrum* strain in Japan. Biocontrol Sci..

[41] Nizam T.M., Binting R.A.A., Saari S.M., Kumar T.V., Muhammad M., Satim H., Yusoff H., Santhanam J. (2016). In Vitro Antifungal Activities against Moulds Isolated from Dermatological Specimens. Malays. J. Med. Sci..

[42] Osborne C.S., Leitner I., Hofbauer B., Fielding C.A., Favre B., Ryder N.S. (2006). Biological, Biochemical, and Molecular Characterization of a New Clinical *Trichophyton rubrum* Isolate Resistant to Terbinafine. Antimicrob. Agents Chemother..

[43] Mukherjee P.K., Leidich S.D., Isham N., Leitner I., Ryder N.S., Ghannoum M.A. (2003). Clinical *Trichophyton rubrum* Strain Exhibiting Primary Resistance to Terbinafine. Antimicrob. Agents Chemother..

[44] Maurya V.K., Kachhwaha D., Bora A., Khatri P.K., Rathore L. (2019). Determination of antifungal minimum inhibitory concentration and its clinical correlation among treatment failure cases of dermatophytosis. J. Fam. Med. Prim. Care.

[45] Dogra S., Pathania S., Rudramurthy S., Narang T., Saikia U. (2018). A prospective study of the epidemiological and clinical patterns of recurrent dermatophytosis at a tertiary care hospital in India. Indian J. Dermatol. Venereol. Leprol..

[46] Dabas Y., Xess I., Singh G., Pandey M., Meena S. (2017). Molecular Identification and Antifungal Susceptibility Patterns of Clinical Dermatophytes Following CLSI and EUCAST Guidelines. J. Fungi.

[47] Rudramurthy S.M., Shankarnarayan S.A., Dogra S., Shaw D., Mushtaq K., Paul R.A., Narang T., Chakrabarti A. (2018). Mutation in the Squalene Epoxidase Gene of *Trichophyton interdigitale* and *Trichophyton rubrum* Associated with Allylamine Resistance. Antimicrob. Agents Chemother..

[48] Bhatia V.K., Sharma P.C. (2014). Epidemiological studies on Dermatophytosis in human patients in Himachal Pradesh, India. SpringerPlus.

[49] Drake L.A., Shear N.H., Arlette J.P., Cloutier R., Danbye F.W., Elewski B.E., Garnis-Jones S., Giroux J.-M., Gratton D., Gulliver W. (1997). Oral terbinafine in the treatment of toenail onychomycosis: North American multicenter trial. J. Am. Acad. Dermatol..

[50] Osborne C.S., Leitner I., Favre B., Ryder N.S. (2005). Amino Acid Substitution in *Trichophyton rubrum* Squalene Epoxidase Associated with Resistance to Terbinafine. Antimicrob. Agents Chemother..

[51] Gregoriou S., Mpali N., Vrioni G., Hatzidimitriou E., Chryssou S.-E., Rigopoulos D. (2020). Epidemiology of Onychomycosis in an Academic Nail Unit in South Greece during a Three-Year Period. Ski. Appendage Disord..

[52] Nasr A., Vyzantiadis T., Patsatsi A., Louka A., Ioakimidou A., Zachrou E., Chavale A., Kalabalikis D., Malissiovas N., Sotiriadis D. (2015). Epidemiology of superficial mycoses in Northern Greece: A 4-year study. J. Eur. Acad. Dermatol. Venereol..

[53] Hayette M.-P., Sacheli R. (2015). Dermatophytosis, Trends in Epidemiology and Diagnostic Approach. Curr. Fungal Infect. Rep..

[54] Badali H., Mohammadi R., Mashedi O., De Hoog G.S., Meis J.F. (2015). In vitro susceptibility patterns of clinically important *Trichophyton* and *Epidermophyton* species against nine antifungal drugs. Mycoses.

[55] Zalacain A., Obrador C., Martinez J.P., Viñas M., Vinuesa T. (2010). Characterization of the antimicrobial susceptibility of fungi responsible for onychomycosis in Spain. Med Mycol..

[56] Jiang Y., Luo W., Verweij P.E., Song Y., Zhang B., Shang Z., Al-Hatmi A.M.S., Ahmed S.A., Wan Z., Li R. (2020). Regional Differences in Antifungal Susceptibility of the Prevalent Dermatophyte *Trichophyton rubrum*. Mycopathologia.

[57] Yenişehirli G., Tunçoğlu E., Yenisehirli A., Bulut Y. (2013). In vitro activities of antifungal drugs against dermatophytes isolated in Tokat, Turkey. Int. J. Dermatol..

[58] Singh J., Zaman M., Gupta A.K. (2007). Evaluation of microdilution and disk diffusion methods for antifungal susceptibility testing of dermatophytes. Med. Mycol..

[59] Ghannoum M.A., Hajjeh R.A., Scher R., Konnikov N., Gupta A.K., Summerbell R., Sullivan S., Daniel R., Krusinski P., Fleckman P. (2000). A large-scale North American study of fungal isolates from nails: The frequency of onychomycosis, fungal distribution, and antifungal susceptibility patterns. J. Am. Acad. Dermatol..

[60] Silva L.B., De Oliveira D., Da Silva B., De Souza R., Da Silva P., Ferreira-Paim K., Silva-Vergara M., Andrade A. (2014). Identification and antifungal susceptibility of fungi isolated from dermatomycoses. J. Eur. Acad. Dermatol. Venereol..

[61] Bueno J.G., Martinez C., Zapata B., Sanclemente G., Gallego M., Mesa A.C. (2009). In vitro activity of fluconazole, itraconazole, voriconazole and terbinafine against fungi causing onychomycosis. Clin. Exp. Dermatol..

[62] Khurana A., Masih A., Chowdhary A., Sardana K., Borker S., Gupta A., Gautam R.K., Sharma P.K., Jain D. (2018). Correlation of In Vitro Susceptibility Based on MICs and Squalene Epoxidase Mutations with Clinical Response to Terbinafine in Patients with Tinea Corporis/Cruris. Antimicrob. Agents Chemother..

[63] Arora P., Sardana K., Kaur R., Goyal R., Ghunawat S. (2018). Is antifungal resistance a cause for treatment failure in dermatophytosis: A study focused on tinea corporis and cruris from a tertiary centre?. Indian Dermatol. Online J..

[64] Singh A., Masih A., Monroy-Nieto J., Singh P.K., Bowers J., Travis J., Khurana A., Engelthaler D.M., Meis J.F., Chowdhary A. (2019). A unique multidrug-resistant clonal Trichophyton population distinct from *Trichophyton mentagrophytes/Trichophyton interdigitale* complex causing an ongoing alarming dermatophytosis outbreak in India: Genomic insights and resistance profile. Fungal Genet. Biol..

[65] Taghipour S., Shamsizadeh F., Pchelin I.M., Rezaei-Matehhkolaei A., Mahmoudabadi A.Z., Valadan R., Ansari S., Katiraee F., Pakshir K., Zomorodian K. (2020). Emergence of Terbinafine Resistant *Trichophyton mentagrophytes* in Iran, Harboring Mutations in the Squalene Epoxidase (*SQLE*) Gene. Infect. Drug Resist..

[66] Tang C., Kong X., Ahmed S.A., Thakur R., Chowdhary A., Nenoff P., Uhrlass S., Verma S.B., Meis J.F., Kandemir H. (2021). Taxonomy of the *Trichophyton mentagrophytes/T. interdigitale* Species Complex Harboring the Highly Virulent, Multiresistant Genotype *T. indotineae*. Mycopathologia.

[67] Kano R., Kimura U., Kakurai M., Hiruma J., Kamata H., Suga Y., Harada K. (2020). *Trichophyton indotineae* sp. nov.: A New Highly Terbinafine-Resistant Anthropophilic Dermatophyte Species. Mycopathologia.

[68] Shaw D., Singh S., Dogra S., Jayaraman J., Bhat R., Panda S., Chakrabarti A., Anjum N., Chowdappa A., Nagamoti M. (2020). MIC and Upper Limit of Wild-Type Distribution for 13 Antifungal Agents against a *Trichophyton mentagrophytes-Trichophyton interdigitale* Complex of Indian Origin. Antimicrob. Agents Chemother..

[69] Hiruma J., Kitagawa H., Noguchi H., Kano R., Hiruma M., Kamata H., Harada K. (2018). Terbinafine-resistant strain of *Trichophyton interdigitale* strain isolated from a tinea pedis patient. J. Dermatol..

[70] Gnat S., Łagowski D., Nowakiewicz A., Dyląg M., Osińska M. (2021). Complementary effect of mechanism of multidrug resistance in *Trichophyton mentagrophytes* isolated from human dermatophytoses of animal origin. Mycoses.

[71] Martinez-Rossi N.M., Bitencourt T.A., Peres N.T.A., Lang E.A.S., Gomes E.V., Quaresemin N.R., Martins M., Lopes L., Rossi A. (2018). Dermatophyte Resistance to Antifungal Drugs: Mechanisms and Prospectus. Front. Microbiol..

[72] Kimura U., Hiruma M., Kano R., Matsumoto T., Noguchi H., Takamori K., Suga Y. (2020). Caution and warning: Arrival of terbinafine-resistant *Trichophyton interdigitale* of the Indian genotype, isolated from extensive dermatophytosis, in Japan. J. Dermatol..

[73] Kakurai M., Harada K., Maeda T., Hiruma J., Kano R., Demitsu T. (2020). Case of tinea corporis due to terbinafine-resistant *Tri-chophyton interdigitale*. J. Dermatol..

[74] Hsieh A., Quenan S., Riat A., Toutous-Trellu L., Fontao L. (2019). A new mutation in the SQLE gene of *Trichophyton mentagrophytes* associated to terbinafine resistance in a couple with disseminated tinea corporis. J. Mycol. Médicale.

[75] Sacheli R., Harag S., Dehavay F., Evrard S., Rousseaux D., Adjetey A., Seidel L., Laffineur K., Lagrou K., Hayette M.-P. (2020). Belgian National Survey on Tinea Capitis: Epidemiological Considerations and Highlight of Terbinafine-Resistant *T. mentagrophytes* with a Mutation on SQLE Gene. J. Fungi.

[76] Järv H., Uhrlass S., Simkin T., Nenoff P., Alvarado Ramirez E., Chryssanthou E., Monod M. (2019). Terbinafine resistant *Trichophyton mentagrophytes* genotype VIII, Indian type, isolated in Finland. J. Fungi.

[77] Burmester A., Hipler U.-C., Hensche R., Elsner P., Wiegand C. (2019). Point mutations in the squalene epoxidase gene of Indian ITS genotype VIII *T. mentagrophytes* identified after DNA isolation from infected scales. Med Mycol. Case Rep..

[78] Łagowski D., Gnat S., Nowakiewicz A., Osińska M., Dyląg M. (2020). Intrinsic resistance to terbinafine among human and animal isolates of *Trichophyton mentagrophytes* related to amino acid substitution in the squalene epoxidase. Infection.

[79] Manoyan M., Sokolov V., Gursheva A., Gabuzyan N., Panin A. (2019). Sensitivity of isolated dermatophyte strains to antifungal drugs in the Russian Federation. J. Fungi.

[80] Curatolo R., Juricevic N., Leong C., Bosshard P.P. (2021). Antifungal susceptibility testing of dermatophytes: Development and evaluation of an optimised broth microdilution method. Mycoses.

[81] Verma S.B. (2018). Emergence of recalcitrant dermatophytosis in India. Lancet Infect. Dis..

[82] Bishnoi A., Vinay K., Dogra S. (2018). Emergence of recalcitrant dermatophytosis in India. Lancet Infect. Dis..

[83] Osborne C.S., Hofbauer B., Favre B., Ryder N.S. (2003). In Vitro Analysis of the Ability of *Trichophyton rubrum* To Become Resistant to Terbinafine. Antimicrob. Agents Chemother..

[84] Ghelardi E., Celandroni F., Gueye S.A., Salvetti S., Senesi S., Bulgheroni A., Mailland F. (2014). Potential of Ergosterol Synthesis Inhibitors To Cause Resistance or Cross-Resistance in *Trichophyton rubrum*. Antimicrob. Agents Chemother..

[85] Khurana A., Sardana K., Chowdhary A. (2019). Antifungal resistance in dermatophytes: Recent trends and therapeutic implications. Fungal Genet. Biol..

[86] Araújo C.R., Miranda K.C., Fernandes O.D.F.L., Soares A.J., Silva M.D.R.R. (2009). In vitro susceptibility testing of dermatophytes isolated in Goiania, Brazil, against five antifungal agents by broth microdilution method. Rev. Inst. Med. Trop. São Paulo.

[87] Arendrup M.C., Jørgensen K.M., Guinea J., Lagrou K., Chryssanthou E., Hayette M.-P., Barchiesi F., Lass-Flörl C., Hamal P., Dannaoui E. (2020). Multicentre validation of a EUCAST method for the antifungal susceptibility testing of microconidia-forming dermatophytes. J. Antimicrob. Chemother..

[88] Baghi N., Shokohi T., Badali H., Makimura K., Rezaei-Matehkolaei A., Abdollahi M., Didehdar M., Haghani I., Abastabar M. (2016). In vitro activity of new azoles luliconazole and lanoconazole compared with ten other antifungal drugs against clinical dermatophyte isolates. Med. Mycol..

[89] Altinbaş R., Özakkaş F., Bariş A., Turan D., Şen S. (2018). In vitro susceptibility of seven antifungal agents against dermatophytes isolated in İstanbul. Turk. J. Med. Sci..

